# Supplementary material for: The genome sequence of meadow thistle, Cirsium dissectum (L.) Hill (Asterales: Asteraceae)
Source: Wellcome Open Res. 2026 Sep 28;11:763. [Version 1] doi: 10.12688/wellcomeopenres.27952.1 (PMC13760146; doi:10.12688/wellcomeopenres.27952.1)
Supplement: Peer review history

# Reviewer report: Reviewer response for version 1

**DOI:** 10.21956/wellcomeopenres.30752.r171463

**Published:** 2026-11-12

**Authors:** Rong Zhang

**Affiliations:** 1 Kunming Institute of Botany Chinese Academy of Sciences, Kunming, Yunnan, China

This study provides a useful chromosome-level genome resource for *Cirsium dissectum*. The sequencing and assembly quality are generally consistent with current standards for chromosome-scale genome assemblies in large-scale biodiversity genomics projects. Please clearly specify which plant tissues (e.g., young leaves) were used for DNA and RNA extraction in the Methods section.

Are the datasets clearly presented in a usable and accessible format, and the assembly and annotation available in an appropriate subject-specific repository?

Yes

Are sufficient details of the sequencing and extraction, software used, and materials provided to allow replication by others?

Yes

Are the rationale for sequencing the genome and the species significance clearly described?

Yes

Are the protocols appropriate and is the work technically sound?

Yes

Reviewer Expertise:

Phylogeny, phylogenomics, and evolution

I confirm that I have read this submission and believe that I have an appropriate level of expertise to confirm that it is of an acceptable scientific standard.

# Reviewer report: Reviewer response for version 1

**Published:** 2026-09-28

**Authors:** Automated Benchmarking

**Affiliations:** 1 Wellcome Sanger Institute, Hinxton, UK

As this article is a Genome Note, Automated Benchmarking has been used to determine whether the reported assembly passes four defined checks established by the Earth BioGenome Project: the raw and assembled data are publicly available, the Earth BioGenome Project three-part metric is met, the k-mer completeness exceeds 90%, and the BUSCO score passes the stated threshold. For Cirsium dissectum (daCirDiss1), all four checks passed. Data available: pass. ENA study PRJEB85034, raw-read accessions ERR14209144, ERR14209145, ERR14224624, and ERR14224625, and assembly accessions GCA_965276805.1 and GCA_965276745.1 are public. Earth BioGenome Project three-part metric: pass. Haplotype 1 is reported as 6.C.Q61, meeting the 6.C.Q40 reference standard. k-mer completeness: pass. The combined haplotype assemblies have 98.62% k-mer completeness, exceeding the Earth BioGenome Project target of 90%. BUSCO: pass. Haplotype 1, which is proposed as the reference assembly for this species, has 97.7% complete BUSCOs (90.8% single-copy and 6.9% duplicated). BUSCO software version: 5.7.1; lineage dataset: eudicots_odb10. It meets the plant-specific target of greater than 90% complete BUSCOs. This decision uses values reported in the Genome Note and public accession records.

Reviewer Expertise:

NA

I confirm that I have read this submission and believe that I have an appropriate level of expertise to confirm that it is of an acceptable scientific standard.
